# A Tailor-Made Mobile App With a Local Cuisine Database for Self-Management of Type 2 Diabetes Mellitus: Randomized Controlled Trial

**DOI:** 10.2196/83685

**Published:** 2025-12-29

**Authors:** Supasuta Wongdama, Wannaporn Paemueang, Chutintorn Sriphrapradang

**Affiliations:** 1 Division of Endocrinology and Metabolism, Department of Medicine, Faculty of Medicine, Ramathibodi Hospital, Mahidol University, 270 Rama VI Road, Ratchatewi, Bangkok, 10400, Thailand, 66 22011647

**Keywords:** Asian, culture, lifestyle, healthy diet, mobile health, mHealth, self-management

## Abstract

**Background:**

There are many mobile apps for diabetes self-management; however, most target Western populations and lack dietary content relevant to Asian contexts. Our mobile app addresses this gap by providing self-care tools and a database of regionally relevant foods.

**Objective:**

This study aimed to evaluate the effectiveness of the app in improving glycemic control and self-care behaviors among outpatients with uncontrolled type 2 diabetes at our hospital.

**Methods:**

We conducted a randomized controlled trial with adults with type 2 diabetes, hemoglobin A_1c_ (HbA_1c_) of >7%, and access to a smartphone. Participants were randomized to an intervention group (daily use of the Rama Diabetes Care app) or a control group (standard care), with all receiving diabetes self-management education and support. The app includes 6 features, notably a nutritional logging system with a verified database of Thai and commonly consumed foods, including Asian and Western dishes, as well as blood glucose monitoring, exercise and medication tracking, symptom screening, and weight logging. The primary outcome was HbA_1c_ level, and secondary outcomes included fasting plasma glucose (FPG), low-density lipoprotein cholesterol, estimated glomerular filtration rate, BMI, self-care behaviors, and user satisfaction with the app. The study was conducted between November 29, 2023, and October 30, 2024.

**Results:**

A total of 129 participants were randomized (intervention: n=64, 49.6%; control: n=65, 50.4%). Participants in the intervention group were younger (mean age 54.6, SD 14.3 years vs 61.9, SD 12.0 years; *P*=.002), whereas baseline HbA_1c_ (mean 9.3%, SD 1.96%) and FPG (mean 179.5, SD 5.9 mg/dL) levels were similar between the groups. Over 6 months, the intervention group showed a greater HbA_1c_ reduction than the control group (mean difference −0.24%), but the difference was not statistically significant (*P*=.13). Among participants aged <65 years, FPG at 6 months was significantly lower in the intervention group (mean difference −29.3 mg/dL; *P*=.03). App satisfaction was rated as moderate.

**Conclusions:**

The mobile app achieved glycemic control comparable to that achieved through standard care, with significant improvement in FPG among participants younger than 65 years. Tailor-made apps integrating regionally relevant dietary content may support effective self-management in type 2 diabetes and warrant further evaluation in larger, long-term studies.

## Introduction

Type 2 diabetes mellitus (T2DM) is a major global health challenge that requires lifestyle modification and often pharmacological therapy. Beyond glycemic control, contemporary management emphasizes patient empowerment through education, behavioral support, and individualized care. The American Diabetes Association highlights the central role of diabetes self-management education and support (DSMES), recommending developmentally and culturally tailored education to enhance knowledge, confidence, and sustainable self-care behaviors [1]. In Thailand, a systematic review by Jerawatana et al [2] found that DSMES participation significantly reduced hemoglobin A_1c_ (HbA_1c_) level by 0.66% within 6 months, underscoring the clinical value of structured education.

Despite its proven benefits, DSMES in Thailand remains predominantly hospital based and resource intensive, limiting accessibility amid the rising prevalence of diabetes. Digital health innovations, particularly mobile health (mHealth) apps, offer scalable solutions to extend DSMES into daily life. The World Health Organization defines mHealth as the use of mobile and wireless technologies to support health objectives [3]. With smartphones now embedded in everyday life, a 2022 systematic review identified 458 mobile apps supporting diabetes medication adherence, with numbers continuing to rise [4]. From the user perspective, one-third of individuals currently use mHealth apps for diabetes self-management, and more than half express interest in future adoption [5]. Moreover, a recent systematic review reported that 11 of 12 studies showed significant HbA_1c_ reductions with mobile app use [6]. However, apps vary widely in design and features, and few incorporate content relevant to Thai users, such as local dietary databases or Thai-language interfaces.

In Thailand, existing tools such as DM Thai Diary for type 1 diabetes and EASYDM for medication adherence [7] address only selected aspects of care, and no comprehensive application for T2DM is available [8]. To address this gap, our institute developed the Rama Diabetes Care app, which provides a comprehensive nutritional database featuring both Thai and international foods and integrates 6 essential self-care domains (nutrition, medication adherence, blood glucose monitoring, physical activity, psychosocial well-being [symptom screening], and weight management) into a single, all-in-one platform. This study evaluated its effectiveness in improving glycemic control and self-care behaviors among patients with uncontrolled T2DM.

## Methods

### Ethical Considerations

All patients provided written informed consent. Patient data were fully anonymized prior to analysis, with access strictly limited to the study team and the software developer. No financial compensation or reimbursement was provided to participants. The study protocol was approved by the Human Research Ethics Committee of the Faculty of Medicine Ramathibodi Hospital, Mahidol University (MURA2023/872). This trial has been registered on ClinicalTrials.gov (NCT06176703).

### App Design and Development

**Figure 1.**
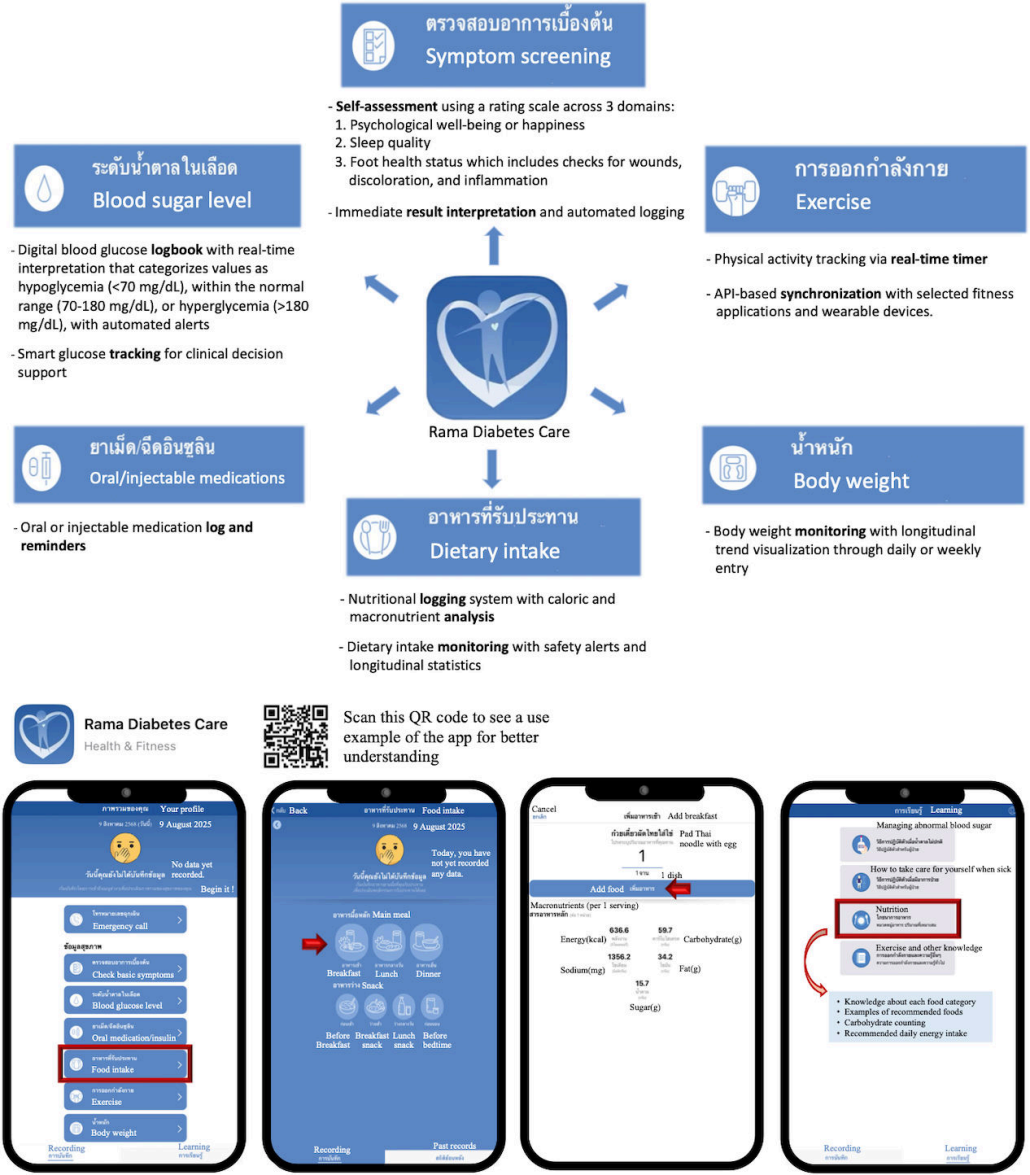
The 6 core features of the Rama Diabetes Care app: symptom screening, blood glucose monitoring, medication logging, dietary intake logging, physical activity tracking, and body weight monitoring. As an example of app use, the app operates through the following steps: (1) tapping the app icon, (2) selecting the desired category (shown in this figure as the “food” category), (3) choosing the meal to be logged, and (4) entering the name of the consumed food item. The app then displays the energy content of the selected food, which can be saved for future reference or modification. API: application programming interface.

The Rama Diabetes Care app (Figure 1) was developed based on DSMES principles to provide comprehensive self-management support for patients with T2DM, including self-monitoring and interpretation of blood glucose, physical activity tracking, nutritional therapy, and other self-care tasks. The app was developed by Vernity in collaboration with the Faculty of Medicine of Ramathibodi Hospital. To ensure reliability and content validity, the app’s content was rigorously reviewed by a multidisciplinary team; specifically, the nutritional database was verified by certified nutritionists, whereas other app components and clinical algorithms were validated by endocrinologists to ensure medical accuracy. The app provides alerts to help patients maintain appropriate blood glucose levels and support digital recording of self-monitoring data, reducing reliance on paper logs. It also delivers educational content to promote effective diabetes self-management. While the app does not offer real-time physician-patient interaction, users can contact the emergency number within the app or reach the diabetes care team through the available channels if questions or urgent issues arise. To strictly comply with the hospital’s cybersecurity protocols, the app operates as a stand-alone platform without direct electronic medical record integration. Instead, it functions as a digital logbook, allowing patients to present visualized data directly from their smartphones to physicians during consultations for immediate treatment adjustments.

The app comprises 6 key features. Nutritional logging is a key feature of the app, incorporating a verified database of Thai and commonly consumed international foods. Nutritional values for international dishes were obtained from the US Department of Agriculture FoodData Central database, whereas nutritional values for Thai dishes were calculated using INMUCAL (version 3.0) and the Nutrition 100 g program, both developed by the Institute of Nutrition, Mahidol University, and subsequently verified by certified nutritionists. The app provides energy (kcal), carbohydrate (g), sodium (mg), fat (g), and sugar (g) information, offering immediate feedback on dietary appropriateness and statistical overviews. Other features include blood glucose monitoring, with alerts indicating whether levels are within the target range (70‐180 mg/dL); medication logging, with reminders and intake recording; exercise tracking with manual or device-synchronized entries; daily weight logging; and symptom screening evaluating quality of life and diabetic foot issues. To support long-term monitoring, the app features comprehensive visualization tools, enabling users to view nutritional and physical activity trends over various timelines (daily to yearly), blood glucose summaries highlighting abnormal readings, and longitudinal weight graphs. Collectively, these features serve as reminders for behavioral modification and provide essential data for optimizing future medical treatment.

The app is compatible with both iOS and Android systems. Technical issues were addressed by the research team in coordination with the developers.

### Recruitment and Data Collection

Patients attending DSMES sessions at the diabetes counseling group in the outpatient department on Tuesday and Thursday mornings were invited to participate in the study. The study was conducted between November 29, 2023, and October 30, 2024. Eligible participants were adults (≥18 years) with T2DM, access to a smartphone and the internet, and no prior use of this app. For patients aged ≥65 years who were not proficient with smartphone use, daily caregivers could operate the app on their behalf, with informed consent obtained from the caregivers. Exclusion criteria included use of continuous glucose monitoring or insulin pumps, pregnancy, or visual or hearing impairments.

Eligible patients were randomly assigned to the intervention or control group using a block of 2 generated by an independent statistician. The investigator performed allocation via a digital concealed system. To prevent data contamination, access to the app, which is not publicly available, was restricted through unique log-in credentials (username and password) generated exclusively by the research team, and participants were strictly instructed not to share these credentials. Participants in the intervention group received training on app use and were encouraged to engage with the app at home. One week after randomization, a follow-up phone call was made to assess adherence and address any app-related issues.

Baseline laboratory data, including HbA_1c_ level, fasting plasma glucose (FPG), estimated glomerular filtration rate, and low-density lipoprotein cholesterol (LDL-c), were retrieved directly from the hospital’s electronic medical record. Data obtained within 3 months before enrollment were permitted. However, in most cases, these values were obtained on the screening day during the participants’ scheduled visits. BMI was calculated from body weight and height measured at screening. Outcomes were assessed at 3 and 6 months after randomization. The primary outcome was HbA_1c_ level. Secondary outcomes included objective measurements (FPG, estimated glomerular filtration rate, LDL-c, and BMI) and subjective outcomes assessed via questionnaires. For these objective measurements, we coordinated with the participants’ attending physicians to ensure that the required tests were ordered during follow-up visits. Assessment bias was minimized by relying on these objective values, which were processed by hospital laboratory staff blinded to group allocation. Subjective outcomes were assessed using validated questionnaires, including the DSMES questionnaire, metabolic equivalent of task score, Global Physical Activity Questionnaire, Generalized Anxiety Disorder–7, Patient Health Questionnaire–9, EQ-5D-5L, and satisfaction with diabetes management. Intervention participants additionally rated their satisfaction with the app at 3 and 6 months using a Likert scale developed by the research team. Finally, to ensure impartiality during the evaluation phase, data analysis was conducted on deidentified data after completion of the 6-month follow-up. Six-month outcomes were analyzed using available data only, and participants with missing outcome data at the 6-month follow-up were excluded from the analysis. The study protocol remained unchanged after trial commencement, with no modifications to eligibility criteria, interventions, or outcome assessments.

### Statistical Analyses

The sample size was calculated using 2 independent means based on the study by Goodarzi et al [9], resulting in a total of 128 participants (64 in each group). Baseline characteristics of the study population were summarized as percentages for categorical variables and as means and SDs or medians and IQRs for continuous variables as appropriate. Baseline HbA_1c_ level was defined as the value measured within 3 months before the DSMES session, whereas follow-up HbA_1c_ values were obtained at physician appointments, allowing for a deviation of up to 1 month from the 3- and 6-month time points.

Comparisons of outcomes between the intervention and control groups at each time point were performed using independent-sample 2-tailed *t* tests for normally distributed data and Mann-Whitney rank sum tests for nonnormally distributed data. Statistical significance was set at *P*<.05. Subgroup analyses were conducted using the same methods, and baseline imbalances were adjusted in outcome analyses as appropriate. All statistical analyses were performed using Stata (version 18.0; StataCorp LLC).

## Results

**Figure 2.**
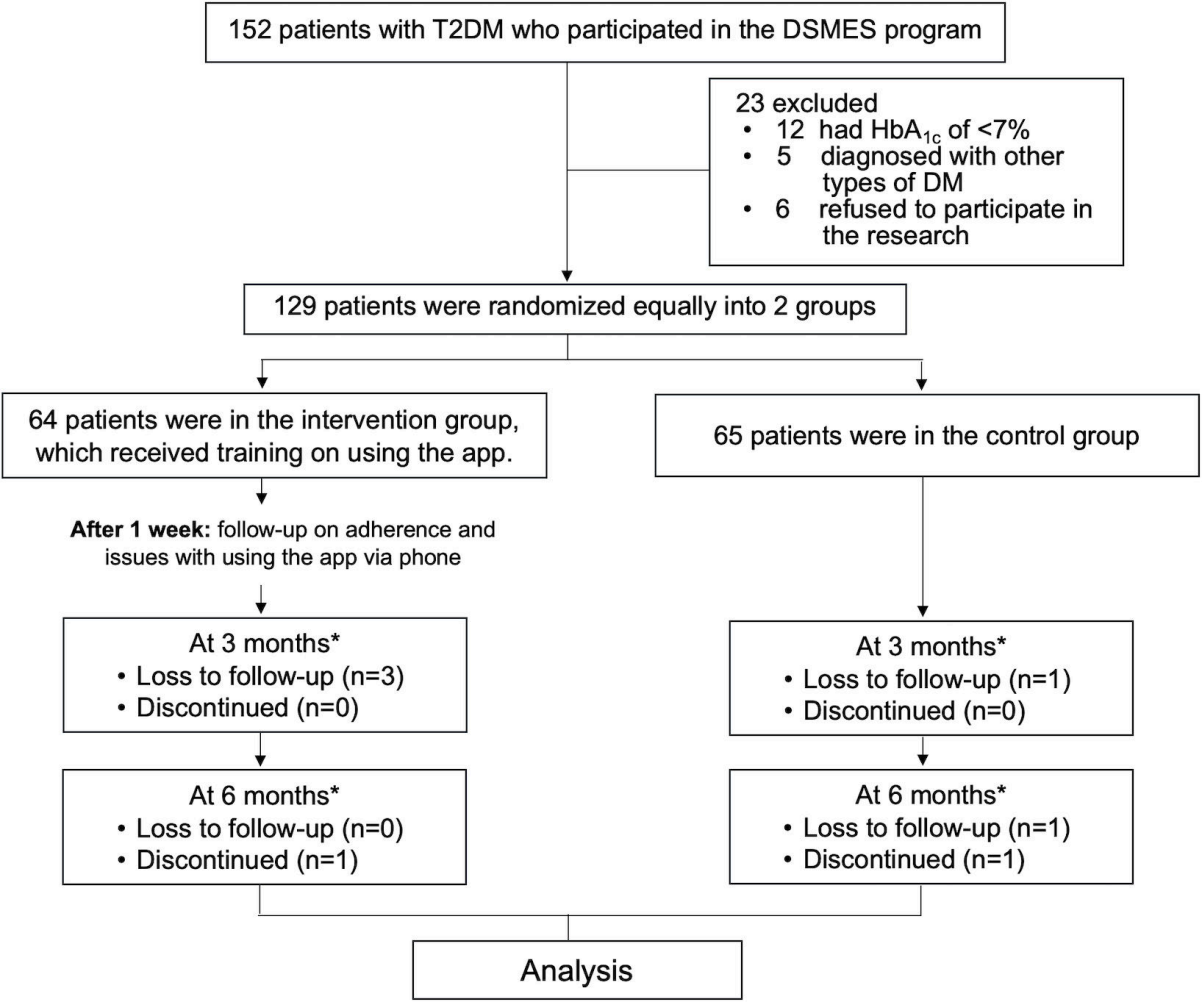
Study flowchart. DM: diabetes mellitus; DSMES: diabetes self-management education and support; HbA_1c_: hemoglobin A_1c_; T2DM: type 2 diabetes mellitus *Assessment included objective assessment (HbA_1c_, low-density lipoprotein cholesterol, estimated glomerular filtration rate, and BMI) and subjective assessment (self-care behaviors via questionnaire and satisfaction of the app; only for the intervention group).

A total of 152 participants from the diabetes counseling group were initially assessed for eligibility (Figure 2), of whom 23 (15.1%) were excluded for not meeting the inclusion criteria, leaving 129 (84.9%) participants (mean age 58, SD 1.2 years; n=74, 57.4% female; mean BMI 27.3, SD 5.2 kg/m^2^; mean duration of diabetes 9.7, SD 0.3 years) who were randomized into the intervention (n=64, 49.6%) and control (n=65, 50.4%) groups. Mean baseline HbA_1c_ levels were 9.3% (SD 2%) in the intervention group and 9.1% (SD 1.6%) in the control group, with no statistically significant difference (*P*=.61). Participants in the intervention group were significantly younger than those in the control group (mean 54.6, SD 14.3 years vs mean 61.9, SD 12.0 years, respectively; *P*=.002; mean age difference of 7 years). Other baseline characteristics, including duration of diabetes and proportion of patients receiving insulin therapy and other medications, were comparable between groups (Table 1).

**Table 1.** Baseline characteristics.

Characteristic	Intervention group (n=64)	Control group (n=65)	*P* value
Age (y), mean (SD)	54.6 (14.3)	61.9 (11.9)	.002
Female participants, n (%)	35 (54.7)	39 (60.0)	.29
Duration of diabetes mellitus (y), median (IQR)	9.2 (1-20)	10.2 (1-22)	.31
Number of diabetic complications^a^, n (%)			.14
0‐1	47 (73.4)	42 (64.6)	
2‐3	16 (25.0)	22 (33.8)	
4‐6	1 (1.6)	1 (1.5)	
Patients treated with insulin, n (%)	44 (68.8)	43 (66.2)	.59
Previously attended a DSMES^b^ program, n (%)	44 (68.8)	49 (75.4)	.15
Education beyond a bachelor’s degree, n (%)	47 (73.4)	32 (49.2)	<.001
Body weight (kg), mean (SD)	73.0 (16.1)	69.8 (18.9)	.30
BMI (kg/m^2^), mean (SD)	27.6 (4.9)	26.9 (5.6)	.52
Fasting plasma glucose (mg/dL), mean (SD)	177.6 (66.0)	181.3 (68.7)	.76
HbA_1c_^c^ (%), mean (SD)	9.3 (2.0)	9.1 (1.6)	.61
LDL-c^d^ (mg/dL), mean (SD)	118.7 (43.4)	99.1 (38.5)	.02
eGFR^e^ (mL per min per 1.73 m^2^), mean (SD)	80.3 (28.8)	73.9 (34.5)	.25
Questionnaires about diabetic self-care			
DSMES score, mean (SD)	59.5 (11.2)	51.3 (12.3)	<.001
MET^f^ score, median (IQR)	202.14 (0-3080)	228.76 (0-2700)	.17
GPAQ^g^ score, median (IQR)	53.6 (0-960)	174.5 (0-3150)	.67
GAD-7^h^ score, median (IQR)	2.4 (0-9)	1.7 (0-8)	.06
QOL^i^ score, mean (SD)	5.9 (1.6)	6.1 (1.3)	.33

a Diabetic complications: diabetic retinopathy, diabetic kidney disease, diabetic neuropathy, and clinical atherosclerotic cardiovascular disease.

b DSMES: diabetes self-management education and support.

c HbA_1c_: hemoglobin A1c.

d LDL-c: low-density lipoprotein cholesterol.

e eGFR: estimated glomerular filtration rate.

f MET: metabolic equivalent of task.

g GPAQ: Global Physical Activity Questionnaire.

h GAD-7: Generalized Anxiety Disorder–7.

i QOL: quality of life.

**Figure 3.**
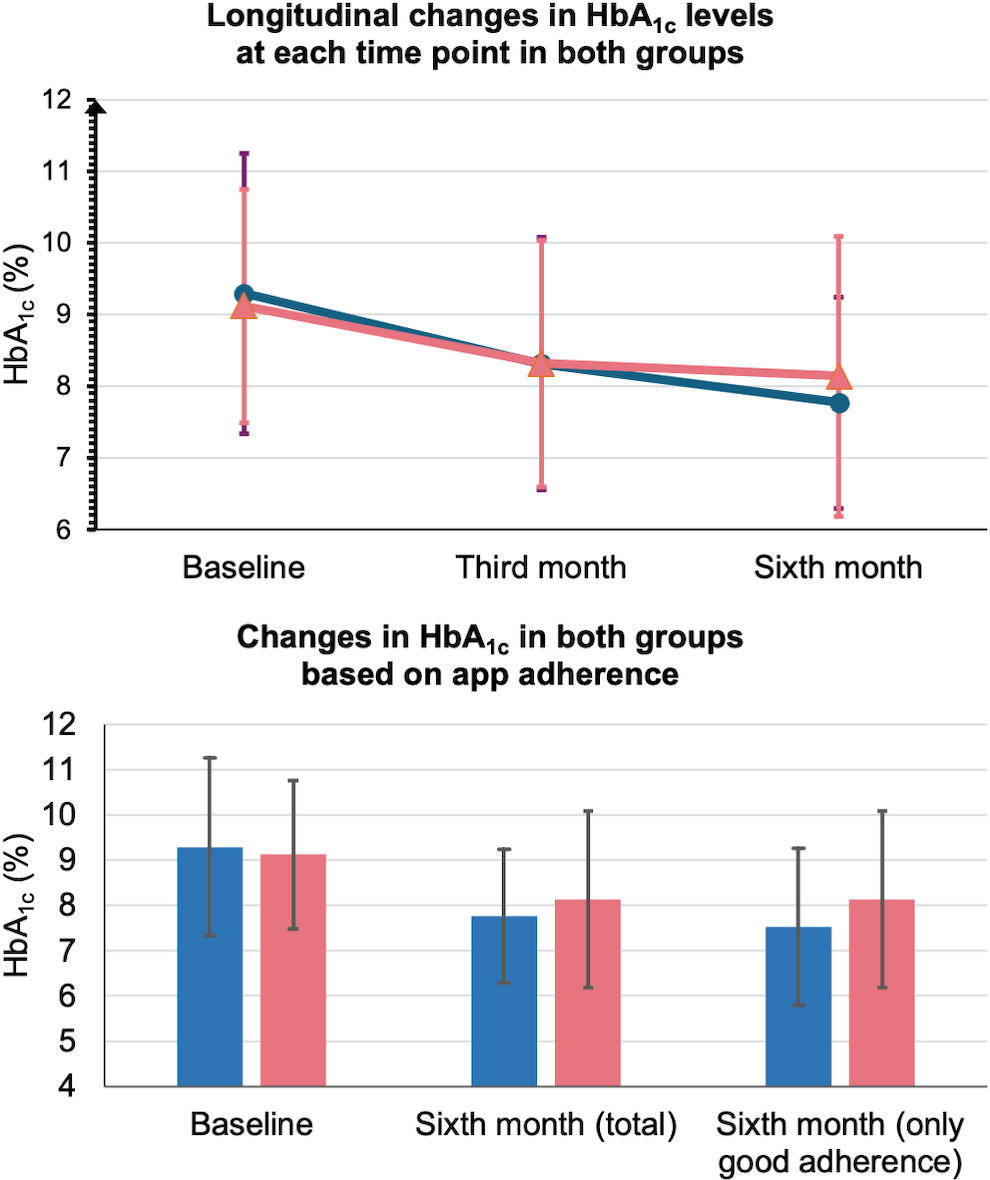
Comparative changes in hemoglobin A_1c_ (HbA_1c_) levels between the intervention and control groups. The blue lines and boxes indicate the intervention group, and the pink lines and boxes indicate the control group.

At 3 months (Figure 3), both the intervention and control groups showed a trend toward reduced HbA_1c_ levels (mean 8.3%, SD 1.8% vs mean 8.3%, SD 1.7%, respectively), consistent with reductions in FPG (mean 142.2, SD 51.7 mg/dL vs mean 159.8, SD 62.6 mg/dL, respectively). Other parameters—including body weight, serum LDL-c, and diabetes-related questionnaire scores—showed only minor changes from baseline, as detailed in Multimedia Appendix 1.

At 6 months, the end of data collection, 4.7% (6/129) of the total participants were lost to follow-up, including 4 in the intervention group and 2 in the control group. In the intervention group, 4.7% (3/64) of the participants discontinued due to difficulties using the app, and 1.6% (1/64) transferred care to another hospital. In the control group, 1.5% (1/65) of the participants were lost to follow-up, and 1.5% (1/65) transferred treatment to another facility. Mean HbA_1c_ levels were 7.8% (SD 1.5%) in the intervention group and 8.1% (SD 1.9%) in the control group, with a nonsignificant difference of 0.24%. FPG was approximately 10 mg/dL lower in the intervention group at 6 months (mean 133.4, SD 43.1 mg/dL vs mean 144.9, SD 67.9 mg/dL), also without statistical significance. Participants in the intervention group exhibited significantly higher DSMES scores from baseline to 6 months. Additional outcomes are detailed in MultimediaAppendices 1  2.

Subgroup analyses were conducted among participants potentially more likely to benefit from the app (Multimedia Appendix 3), including those aged <65 years, with diabetes duration of >10 years, educational level above a bachelor’s degree, and BMI of >25 kg/m^2^. Notably, a statistically significant difference in FPG was observed specifically in participants aged <65 years. In the subgroup of participants aged <65 years, mean FPG at 6 months was 178.8 (SD 66.2) mg/dL in the intervention group and 193.1 (SD 75.5) mg/dL in the control group, with a statistically significant mean difference of 29.3 mg/dL. Average adherence to the app, defined as the proportion of days with recorded blood glucose measurements, was 55.8%. Among participants with good adherence (>50%), the mean 6-month HbA_1c_ level was 7.5% (SD 1.7%) in the intervention group and 8.1% (SD 1.9%) in the control group (*P*=.51). Mean satisfaction scores for the app were 23 and 24 out of 35 at 3 and 6 months, respectively, indicating moderate satisfaction. Item analysis revealed that participants rated the app’s benefits for diabetes self-management highest among all domains. Regarding safety, no app-related adverse events were observed during the study period. There were no reports of severe hypoglycemia or hospitalizations due to hyperglycemic crises resulting from the use of the app.

## Discussion

### Principal Findings

The culturally tailored, Thai-language Rama Diabetes Care app, incorporating regional foods and Thai fonts, is feasible and can support diabetes self-management by providing behavioral alerts and accessible educational content. The inclusion of a verified database covering Thai dishes and specific ingredients (eg, chili paste or *nam phrik*) ensures that the app is highly practical for Thai users. This feature serves as a tool for dietary reflection, allowing patients to assess nutritional value before consumption and develop mindful eating habits that support glycemic control. Furthermore, unlike paper-based methods, the digital interface offers convenience and real-time accessibility, encouraging consistent self-monitoring. In this randomized controlled trial, both groups exhibited reductions in HbA_1c_ level at 6 months, with a greater, though not statistically significant, decrease observed in the app group. FPG was lower in younger intervention participants, and DSMES scores were consistently higher in the app group throughout follow-up.

The modest, nonsignificant reduction in HbA_1c_ level observed in this study is consistent with findings from previous research on DSMES. The approximately 1% HbA_1c_ level reduction in the control group receiving DSMES alone aligns with results of prior studies that have reported reductions of 0.66% [2] and 1.5% [10] at 6 months. Variations across studies may be attributable to factors such as physician-directed medication adjustments and baseline patient motivation. Intervention groups using apps in past systematic reviews showed variability in glycemic responses, often influenced by differences in app design, user engagement, and participant characteristics [6,11-15]. For example, the BlueStar diabetes app—the first Food and Drug Administration–approved mobile prescription therapy in the United States—showed no significant HbA_1c_ level reduction in a multicenter pragmatic trial in Canada, likely due to the main barrier of low app use [16]. In our study, although the app demonstrated a downward trend in HbA_1c_ level beyond 6 months, the relatively short follow-up period may have limited the ability to detect statistically significant differences. Overall, participants expressed moderate satisfaction with the app. Users specifically appreciated the immediate feedback and safety alerts provided by the app, particularly regarding the appropriateness of their dietary choices and capillary blood glucose levels. Notably, the highest satisfaction ratings were driven by the perceived benefits of the app in supporting effective diabetes self-management. User feedback also indicated interest in longer app use, suggesting that extended engagement could yield more pronounced improvements. These findings highlight app adherence as a critical determinant of efficacy and may partially explain the modest outcomes observed in this trial. In this study, adherence greater than 50% was not associated with a statistically significant reduction in HbA_1c_ level, suggesting that higher levels of adherence may be necessary to achieve clinical benefits. Further investigation is warranted.

Subgroup analysis revealed that participants younger than 65 years using the app had lower FPG, potentially reflecting greater motivation and proficiency with technology. In contrast, no differences in glycemic control were observed in subgroups defined by BMI of ≥25 kg/m^2^, diabetes duration of ≥10 years, or education above a bachelor’s level. Evidence from a systematic review of 28 studies indicates that younger, more technology-savvy individuals are more likely to engage effectively with mHealth apps [17]. Similarly, a prospective cohort study in China found that patients younger than 40 years with newly diagnosed T2DM experienced greater HbA_1c_ level reductions and improved self-monitoring and physician-patient communication when using a diabetes app [18]. These findings suggest that mobile apps may confer greater benefits in certain populations, highlighting the challenge of designing apps that are usable and effective across diverse user groups.

In addition to glycemic outcomes, this study evaluated patient-reported outcome measures (PROMs), which, according to the US Food and Drug Administration guidance for medical product development, involve questionnaires that allow patients to report on their physical and psychosocial health as well as their quality of life [19]. There is prior evidence suggesting that mHealth interventions can improve both HbA_1c_ and quality of life while reducing diabetes-related distress [20]. App users exhibited higher PROM scores than controls, although the differences were not statistically significant. This may reflect limited emphasis of the app on quality of life domains such as mental health support. Short-term follow-up may also limit detection of meaningful changes, and baseline psychological status could influence responses. Future iterations of the app should integrate features targeting PROMs to more comprehensively support patient-centered outcomes.

This study has several strengths. Its randomized controlled trial design allowed for rigorous evaluation, with comprehensive collection of both objective and subjective outcomes—the latter being particularly valuable for assessing digital health interventions. Follow-up was robust, with less than 10% (6/129, 4.7%) participant attrition. Importantly, this study demonstrates the feasibility and potential efficacy of the Rama Diabetes Care app, a comprehensive, Thai-language platform featuring extensive nutritional data on local foods, thereby reducing usability barriers for Thai patients with T2DM. Distinguishing itself from existing tools such as Carbs App and DM Thai Diary that focus primarily on carbohydrate counting, our app offers a verified database of over 500 items with complete nutritional profiles (energy, sodium, fat, and sugar). These comprehensive data are clinically vital for holistic management, allowing patients to control not just blood glucose but also body weight, blood pressure, and lipid profiles. Consistent with prior research, culturally adapted digital interventions, such as Spanish-language programs in the United States, have been shown to improve glycemic control in users compared to nonusers [21]. Moreover, unlike specialized tools such as mySugr (focused on glucose monitoring) or EASYDM (focused on medication), our app consolidates 6 essential self-care features into a single “all-in-one” platform. This design streamlines the user experience, enabling holistic self-management without the burden of switching between multiple apps.

Limitations should also be noted. The app did not include features such as patient coaching or real-time patient-health care provider communication, which could further enhance glycemic outcomes. Incorporating these features could enhance the app’s effectiveness in glycemic control, as demonstrated in many previous studies [21,22]. Suboptimal adherence likely contributed to the modest effects observed in this study. Future app iterations may benefit from incorporating engagement strategies such as automated notifications, reward systems, and culturally relevant prompts. Sustaining an mHealth platform also requires continuous updates, user-centered design, simplified data entry (eg, photo-based inputs), and robust data security [6]. A study conducted in China found that, among all app domains, patient ratings were lowest for engagement [23]. Additionally, adequate funding is essential for maintenance, and scaling the app for nationwide use may necessitate government support to benefit users broadly. Finally, to maximize clinical impact, future development should focus on educational content that promotes meaningful behavior change, as well as integration of patient-reported outcomes.

### Conclusions

A culturally tailored, Thai-language mobile app with a local cuisine database reduced HbA_1c_ levels more than DSMES alone at 6 months, although the difference was not statistically significant. The intervention also significantly lowered FPG in participants younger than 65 years, potentially reflecting greater motivation and proficiency in using digital self-management tools in this group.

## Supplementary material

10.2196/83685 Multimedia Appendix 1 Primary outcome of the study.

10.2196/83685 Multimedia Appendix 2 Secondary outcomes of the study.

10.2196/83685 Multimedia Appendix 3 Subgroup analysis.

10.2196/83685 Checklist 1 CONSORT-eHEALTH checklist (V 1.6.1).

## Abbreviations

DSMES: diabetes self-management education and support
FPG: fasting plasma glucose
HbA1c: hemoglobin A1c
LDL-c: low-density lipoprotein cholesterol
mHealth: mobile health
PROM: patient-reported outcome measure
T2DM: type 2 diabetes mellitus
