# Supplementary material for: A Tailor-Made Mobile App With a Local Cuisine Database for Self-Management of Type 2 Diabetes Mellitus: Randomized Controlled Trial
Source: JMIR Diabetes. 2025 Dec 29;10:e83685. doi: 10.2196/83685 (PMC12747420; doi:10.2196/83685)
Supplement: Multimedia Appendix 1 [file diabetes-v10-e83685-s001.docx]

| Primary outcome | Baseline | | | 3^rd^ month | | | 6^th^ month | | |
| --- | --- | --- | --- | --- | --- | --- | --- | --- | --- |
|  | Intervention | Control | *P* | Intervention | Control | *P* | Intervention | Control | *P* |
| HbA1c | 9.3 ± 1.9 | 9.1 ± 1.6 | .61 | 8.3 ± 1.8 | 8.3 ± 1.7 | .83 | 7.8 ± 1.5 | 8.1 ± 1.9 | .07 |
| $\Delta$ HbA1c | -0.2 | | .60 | 0.01 | | .97 | 0.24 | | .44 |
| Reduction of HbA1c from baseline | - | | - | 0.9 ± 0.2 | 0.8 ± 0.3 | .09 | 1.4 ± 0.2 | 0.9 ± 0.3 | .13 |

**Multimedia Appendix 1**

Primary outcome of the study

$\Delta$ : Value differences (control group − intervention group)
