# Supplementary material for: A Tailor-Made Mobile App With a Local Cuisine Database for Self-Management of Type 2 Diabetes Mellitus: Randomized Controlled Trial
Source: JMIR Diabetes. 2025 Dec 29;10:e83685. doi: 10.2196/83685 (PMC12747420; doi:10.2196/83685)
Supplement: Multimedia Appendix 2 [file diabetes-v10-e83685-s002.docx]

**Multimedia Appendix 2**

Secondary outcomes of the study

| Secondary outcomes | Baseline | | | 3^rd^ month | | | 6^th^ month | | | |
| --- | --- | --- | --- | --- | --- | --- | --- | --- | --- | --- |
|  | Intervention | Control | *P* | Intervention | Control | *P* | Intervention | Control | *P* |  |
| FPG (mg/dL) | 177.6 ± 65.9 | 181.3 ± 68.7 | .76 | 142.2 ± 51.7 | 159.8 ± 62.6 | .09 | 133.4 ± 43.1 | 144.9 ± 67.9 | .27 |  |
| $\Delta$ FPG (mg/dL) | 3.7 | | .73 | 17.1 | | .11 | 9.2 | | .39 |  |
| BW (kg) | 73.0 ± 16.1 | 69.8 ± 18.9 | .30 | 72.4 ± 15.8 | 71.2 ± 19.2 | .69 | 71.7 ± 15.7 | 70.6 ± 19.1 | .74 |  |
| $\Delta$ BW (kg) | -3.2 | | .29 | -2.4 | | .44 | -2.9 | | .34 |  |
| BMI (kg/m^2^) | 27.6 ± 4.9 | 26.9 ± 5.6 | .51 | 27.5 ± 4.8 | 27.3 ± 5.6 | .84 | 27.4 ± 5.1 | 27.3 ± 5.7 | .92 |  |
| $\Delta$ BMI (kg/m^2^) | -0.6 | | .52 | -0.6 | | .50 | -0.7 | | .43 |  |
| LDL-c (mg/dL) | 118.7 ± 43.4 | 99.1 ± 38.5 | .02 | 108.8 ± 40.5 | 107.5 ± 42.3 | .87 | 99.6 ± 35.2 | 93.7 ± 37.2 | .43 |  |
| $\Delta$LDL-c (mg/dL) | -17.8 | | .02 | -1.2 | | .87 | -4.3 | | .58 |  |
| eGFR  (ml/min/1.73 m^2^) | 80.3 ± 28.8 | 73.9 ± 34.5 | .25 | 78.9 ± 29.3 | 71.4 ± 35.3 | .21 | 77.8 ± 30.9 | 70.5 ± 34.3 | .23 |  |
| $\Delta$ eGFR  (ml/min/1.73 m^2^) | -17.8 | | .02 | -1.2 | | .88 | -4.3 | | .58 |  |
| Diet (points) | 26.4 ± 5.1 | 23.4 ± 6.0 | .003 | 32.4 ± 5.8 | 29.8 ± 5.2 | .01 | 35.2 ± 5.6 | 32.9 ± 5.2 | .02 |  |
| $\Delta$ Diet (points) | -2.9 | | .002 | -2.7 | | .006 | -2.5 | | .013 |  |
| Monitor (points) | 16.3 ± 4.6 | 13.2 ± 3.6 | <.001 | 19.9 ± 4.2 | 16.9 ± 3.2 | <.001 | 20.7 ± 4.3 | 18.1 ± 3.4 | <.001 |  |
| $\Delta$ Monitor (points) | -3.2 | | <.001 | -2.9 | | <.001 | -2.6 | | <.001 |  |
| Physical (points) | 6.1 ± 1.4 | 5.6 ± 1.3 | .04 | 7.4 ± 1.5 | 6.9 ± 1.4 | .08 | 8.0 ± 1.3 | 7.3 ± 1.2 | .002 |  |
| $\Delta$ Physical (points) | -3.2 | | <.001 | -2.9 | | <.001 | -2.6 | | <.001 |  |
| Regimen (points) | 10.7 ± 2.6 | 9.2 ± 2.7 | .001 | 13.0 ± 1.9 | 11.5 ± 2.2 | .0001 | 13.5 ± 1.9 | 12.2 ± 2.2 | <.001 |  |
| $\Delta$Regimen (points) | -1.6 | | <.001 | -1.5 | | <.001 | -1.4 | | <.001 |  |
| DSMES (points) | 59.5 ± 11.2 | 51.3 ± 12.3 | <.001 | 69.3 ± 18.6 | 62.1 ± 17.2 | .03 | 72.6 ± 21.2 | 62.9 ± 24.1 | .02 |  |
| $\Delta$ DSMES (points) | -8.2 | | .009 | -7.1 | | .024 | -9.7 | | .002 |  |
| MET (points) | 202.1  (0, 3080) | 228.8  (0, 2700) | .17 | 937.3  (0, 4410) | 809.2  (0, 4095) | .87 | 849.9 (0, 3360) | 791.2  (0, 4620) | .42 |  |
| $\Delta$ MET (points) | 26.6 | | .86 | -123.5 | | .40 | -50.4 | | .74 |  |
| GPAQ (points) | 53.6  (0, 960) | 174.5  (0, 3150) | .67 | 455.3  (0, 5040) | 225.8  (0, 3150) | .03 | 293.3  (0, 2850) | 172.2  (0, 2700) | .06 |  |
| $\Delta$ GPAQ (points) | 120.9 | | .24 | -229.9 | | .03 | -139.6 | | .19 |  |
| GAD (points) | 2.4 (0, 9) | 1.7 (0, 8) | .06 | 1.0 (0, 7) | 0.8 (0, 7) | .92 | 0.6 (0, 7) | 0.6 (0, 7) | .93 |  |
| $\Delta$ GAD (points) | -0.7 | | .02 | -0.2 | | .49 | 0.1 | | .88 |  |
| QOL (points) | 5.9 ± 1.6 | 6.1 ± 1.3 | .33 | 7.5 ± 1.5 | 7.2 ± 1.6 | .29 | 8.3 ± 1.5 | 7.8 ± 1.5 | .06 |  |
| $\Delta$ QOL (points) | 0.2 | | .02 | -0.2 | | .50 | -0.4 | | .88 |  |
| App’s satisfaction | 1.6 ± 0.6 | 1.9 ± 0.5 |  | 23.2 ± 7.7 | NA |  | 24.2 ± 7.3 | NA |  |  |

$\Delta$ : Value differences (control group − intervention group); ( ) : (min, max)

Abbreviations: BMI, body mass index; BW, body weight; DM, diabetes mellitus; DSMES, diabetes self-management education and support; eGFR, estimated glomerular filtration rate; FPG, fasting plasma glucose; GAD, generalized anxiety disorder; GPAQ, global physical activity questionnaire; LDL-c, low-density lipoprotein cholesterol; MET, metabolic equivalent of task; QOL, quality of life.
