# Supplementary material for: A Tailor-Made Mobile App With a Local Cuisine Database for Self-Management of Type 2 Diabetes Mellitus: Randomized Controlled Trial
Source: JMIR Diabetes. 2025 Dec 29;10:e83685. doi: 10.2196/83685 (PMC12747420; doi:10.2196/83685)
Supplement: Multimedia Appendix 3 [file diabetes-v10-e83685-s003.docx]

**Multimedia Appendix 3**

Subgroup analysis

| **HbA1c (%)** | **Duration of DM**  **>10 years**  **n = 68** | | **Age <65 years**  **n = 84** | | **Education beyond**  **a bachelor’s degree**  **n = 79** | | **BMI**  **> 25 kg/m^2^**  **n = 78** | |
| --- | --- | --- | --- | --- | --- | --- | --- | --- |
|  | **Intervention**  **n = 31** | **Control**  **n = 37** | **Intervention**  **n = 46** | **Control**  **n = 38** | **Intervention**  **n = 47** | **Control**  **n = 32** | **Intervention**  **n = 43** | **Control**  **n = 35** |
| Baseline | 9.2 ± 0.3 | 9.0 ± 0.3 | 9.4 ± 0.3 | 9.5 ± 0.3 | 8.9 ± 0.3 | 9.2 ± 0.3 | 9.4 ± 0.3 | 9.5 ± 0.3 |
| 6^th^ month | 7.9 ± 0.3 | 8.4 ±0.3 | 7.9 ± 0.3 | 8.4 ± 0.3 | 7.6 ± 0.3 | 8.0 ± 0.3 | 8.1 ± 0.3 | 8.6 ± 0.3 |
| *P*-value | .26 | | .13 | | .24 | | .09 | |
| Mean  difference  at 6 months | 0.48 | | 0.54 | | 0.36 | | 0.53 | |
| *P*-value of mean difference | .26 | | .16 | | .37 | | .18 | |

| **FPG**  **(mg/dL)** | **Duration of DM**  **>10 years**  **n = 68** | | **Age**  **<65 years**  **n = 84** | | **Education beyond**  **a bachelor’s degree**  **n = 79** | | **BMI**  **>25 kg/m^2^**  **n = 78** | |
| --- | --- | --- | --- | --- | --- | --- | --- | --- |
|  | **Intervention**  **n = 31** | **Control**  **n = 37** | **Intervention**  **n = 46** | **Control**  **n = 38** | **Intervention**  **n = 47** | **Control**  **n = 32** | **Intervention**  **n = 43** | **Control**  **n = 35** |
| Baseline | 190.1±74.2 | 169.5±48.8 | 178.8±66.2 | 193.1±75.5 | 168.6±57.1 | 194.9±83.7 | 181.0±73.5 | 182.6±58.6 |
| 6^th^ month | 129.9±10.8 | 140.5±10.8 | 136.9±8.9 | 166.2±9.8 | 134.2±8.8 | 150.9±10.7 | 135.6±9.6 | 159.6±10.1 |
| p-value | .41 | | .03 | | .19 | | .18 | |
| Mean  difference  at 6 months | 10.6 | | 29.3 | | 16.8 | | 24.1 | |
| *P*-value of mean difference | .47 | | .03 | | .22 | | .08 | |

Abbreviations: BMI, body mass index; DM, diabetes mellitus; FPG, fasting plasma glucose; HbA1c, hemoglobin A1c.
